# Bibliometric analysis of the current status and trends on medical hyperspectral imaging

**DOI:** 10.3389/fmed.2023.1235955

**Published:** 2023-09-19

**Authors:** Sijia Jiang, Ding Ma, Xin Tan, Mingyu Yang, Qingbin Jiao, Liang Xu

**Affiliations:** Changchun Institute of Optics, Fine Mechanics and Physics, Chinese Academy of Sciences, Changchun, Jilin,China

**Keywords:** hyperspectral imaging (HSI), medical applications, bibliometrics, research status, development trends

## Abstract

Hyperspectral imaging (HSI) is a promising technology that can provide valuable support for the advancement of the medical field. Bibliometrics can analyze a vast number of publications on both macroscopic and microscopic levels, providing scholars with essential foundations to shape future directions. The purpose of this study is to comprehensively review the existing literature on medical hyperspectral imaging (MHSI). Based on the Web of Science (WOS) database, this study systematically combs through literature using bibliometric methods and visualization software such as VOSviewer and CiteSpace to draw scientific conclusions. The analysis yielded 2,274 articles from 73 countries/regions, involving 7,401 authors, 2,037 institutions, 1,038 journals/conferences, and a total of 7,522 keywords. The field of MHSI is currently in a positive stage of development and has conducted extensive research worldwide. This research encompasses not only HSI technology but also its application to diverse medical research subjects, such as skin, cancer, tumors, etc., covering a wide range of hardware constructions and software algorithms. In addition to advancements in hardware, the future should focus on the development of algorithm standards for specific medical research targets and cultivate medical professionals of managing vast amounts of technical information.

## 1. Introduction

Medical imaging has revolutionized the field of medicine, bringing forth significant advancements. So far, medical imaging equipment based on magnetic resonance imaging (MRI), ultrasound, radiography (X-ray, CT) and other technologies has been widely used in the medical field. However, certain limitations such as high costs and potential harm to the human body (such as radiation damage) may impede its further progress in healthcare (1, 2). Therefore, the development of effective, low-cost, non-invasive medical imaging technology will be an important challenge for researchers around the world (3).

Hyperspectral imaging (HSI), also known as spectrometer (4), is a promising emerging optical technology that combines optical imaging and spectral analysis. Originated from remote sensing, it has been explored by NASA for applications in various fields (5), and has received widespread attention in environmental monitoring (6), vegetation research (7), food inspection (8–10), agricultural analysis (11, 12) and other fields. The development of HSI has made it possible to apply in the field of medicine. HSI is a non-invasive and non-ionizing technology that rapidly acquires and analyzes relevant information in its supporting fields (13). The information carried by the reflected, fluorescent and transmitted light captured by HSI from substances has been extensively studied in the medical field and demonstrated to be effective for disease diagnosis, image-guided surgery, and medicine (14, 15). For instance, several studies have demonstrated the potential of HSI for non-invasive detection of haemoglobin, platelets, total bilirubin, and cutaneous wounds by extracting spectral features through 3D convolutional neural network, support vector machine (SVM), and other algorithms (16–21). Medical hyperspectral imaging (MHSI) has emerged as a prominent research area, with scholars continuously addressing challenges related to enhancing the capabilities of feature extraction, automatic recognition, and recognition accuracy.

Bibliometrics refers to the scientific methodology of quantitatively analyzing the corresponding knowledge system using mathematical and statistical techniques. It is employed for the analysis, comparison, and quantification of literature in order to comprehend the development and trends of scientific research. A few studies have used bibliometrics to analyze the application of HSI technology in remote sensing (22, 23), analyze the development and hot spots of radiology, nuclear medicine, medical imaging and other medical fields (24, 25). Few studies reviewed the application of HSI technology in the medical field with the method of bibliometrics. The advancement of technology necessitates a comprehensive evaluation to foster continuous innovation. Historically, scholars have conducted numerous studies on MHSI technology, however, there has been limited systematic review and organization of its research progress. While a few scholars have undertaken reviews in this field, an overall quantitative visual analysis is still lacking. In order to narrow the research gap, we use historical bibliometrics to analyze the dynamics of literary production and find out the historical roots statistically (26, 27). Therefore, in this study, scientific metrology research has been conducted based on the historical background, achievements, past and current development trends of the field to determine expected goals and potential for further advancement. This will help identify research gaps or technologies that require improvement in order to accelerate the commercialization and practical application of HSI technology in the medical field.

The study utilizes the core collection of Web of Science (WOS) database as the data source and employs bibliometric methods to construct a knowledge graph. By analyzing the development trends in this field from 1992 to present, the acquired data will facilitate determining scientific research directions and provide scholars with a comprehensive understanding of the field’s current state. Providing a foundational basis and guiding significance for the sustainable development of MHSI technology.

## 2. Materials and methods

### 2.1. Samples selection

The WOS database comprises indexed journals, conference proceedings, etc. (28–30), developed by Thomson Reuters, it boasts a superior scientific search engine and the most relevant databases for retrieving data (31, 32). And most importantly, the results output from the WOS database are compatible with bibliometric analysis software and can be used as input data for analysis. Therefore, this study selected the core collection of WOS database for research, using advanced search, the search formula was “TS = hyperspectral.” Subsequently, refine the search results and manually select all categories pertaining to medicine within the WOS category, including Radiology Nuclear Medicine Medical Imaging, Engineering Biomedical, Surgery, Ophthalmology, Oncology, Neurosciences, Dermatology, Pharmacy Pharmacy or Cell Biology, and so on. The searching time is July 17, 2023, and a total of 2,274 results were obtained, covering the time span of 1992–2023. The results were set as a “marked list” in the WOS database, and exported in plain text file format to avoid daily updates of the WOS database. Subsequently, we use the built in function of WOS to extract information such as title, author, affiliated unit, citation count, keywords, etc. as the benchmark for bibliometrics analysis.

### 2.2. Methods

Bibliometrics is a potent and valuable method for visualizing and analyzing information, capable of capturing the overall development of a research field over time (33). The software tools VOSviewer and CiteSpace enable the generation of relevant data maps, clusters, burst, centrality measures, link strengths, and other informative outputs from input literature. These powerful analytical tools facilitate comprehensive analysis (34, 35). In summary, this study focuses on MHSI technology and conducts a systematic review and analysis of relevant literature. Preliminary statistical analysis was conducted based on the search results from the WOS database, followed by in-depth analysis and visualization of 2,274 relevant literature using software VOSviewer (1.6.8) and CiteSpace (6.2.R2). The scientific analysis indexes in this study are: (1) development trends, (2) country distribution, (3) core authors, (4) institutional distribution, (5) prolific journals, (6) citation analysis, and (7) research hotspots. Some parameters are used to describe the above indexes (36).

- a) Betweenness centrality (BC)

BC is used to evaluate the links between nodes, which represent research subjects such as authors and institutions, with values typically ranging from 0 to 1. A higher BC indicates a more significant node, closer connections with other research subjects, and relatively greater opportunities for collaboration. Researchers can assess their own and team’s BC to understand their influence and position in the field.

- b) Burst

Burst indicates when a certain subject (viewpoint, keyword, literature, etc.) has become a hot topic, and when it has gained attention, achieving a surge in attention in the field.

- c) Citation counts (CC)

CC represents the number of times a subject has been cited since its publication, including self-citation, which to some extent signifies the subject’s impact in the field (36, 37).

- d) Cluster

Cluster represents a group of closely related nodes, where they congregate to identify authors who collaborate closely and keywords that frequently co-occur. The cluster labeled as “#0” denotes the strongest category comprising elements with the highest degree of similarity to one another (30, 36).

- e) Citation frequency (CF)

CF is the ratio of CC to publication time, which also represents the value it provides in the field.

Finally, the bibliometric analysis results and research progress are summarized, relevant research content is summarized, and the future development directions are prospected. This provides a data foundation and guiding significance for the scientific and advancement of MHSI technology.

## 3. Results

### 3.1. Bibliometric analysis

The indexes and parameters mentioned in the Methods are analyzed as the results of bibliometrics in this section, and the following data is based on these 2,274 results.

#### 3.1.1. Development trends

**Figure 1:**
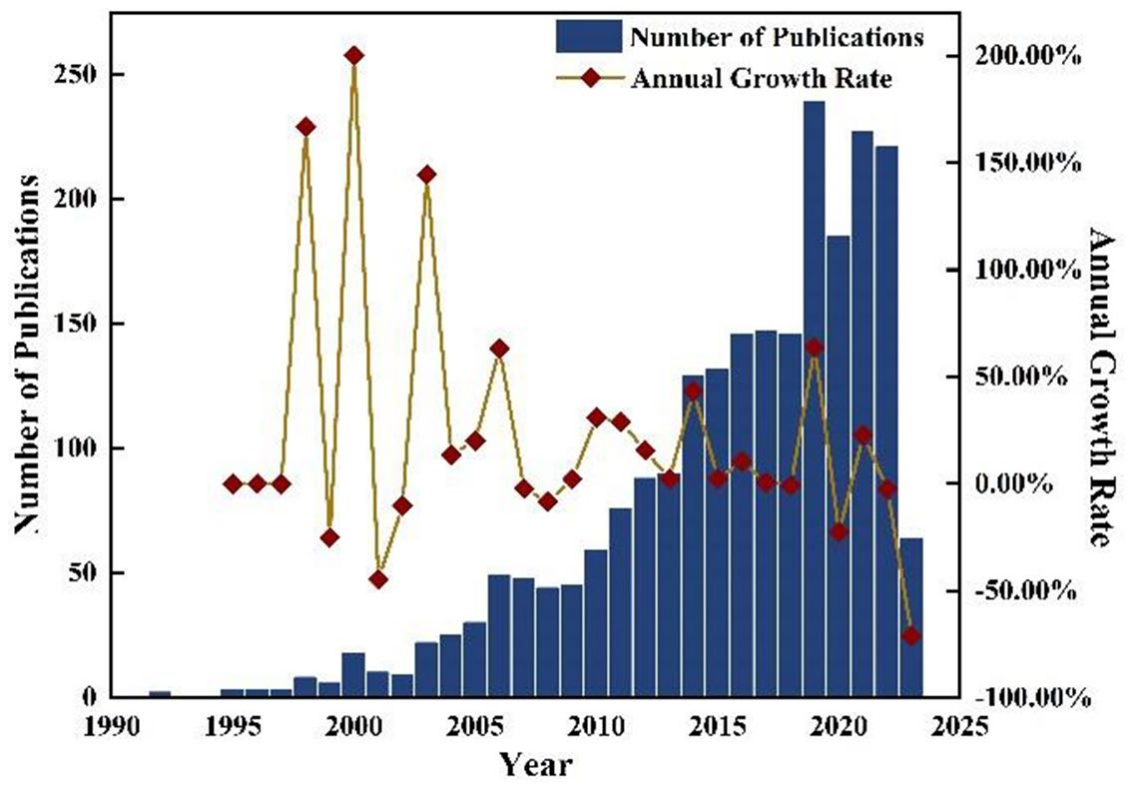
Number of publications and annual growth rate from 1992 to 2023.

In the 1980s, HSI technology commenced its study at the Jet Propulsion Laboratory of the California Institute of Technology in the United States and subsequently underwent rapid development (38). The increase in spectral resolution has enabled the detection and classification of image features based on their spectral characteristics (39). Broadening the surgeon’s field of view would represent a significant breakthrough (40). Algorithms and image processing methods pertaining to multispectral and hyperspectral imaging techniques have been developed, leading to the gradual expansion of this technology in the field of medicine for studying anatomy, physiology, and pathology. The proposal of MHSI has sparked a significant surge in its development, as depicted in Figure 1. This development can be primarily categorized into two stages: 1992–2009 (a stage characterized by slow progress) and 2010-present (a stage marked by rapid advancement). As depicted in Figure 1, the number of articles published in 2019 was 239, has increased nearly fourfold compared to the peak phase of publication in 2006, signifying a recent surge and heightened attention within the field of MHSI. The annual growth rate of the publications is also illustrated in Figure 1 as well.

#### 3.1.2. Country/region distribution

**Figure 2:**
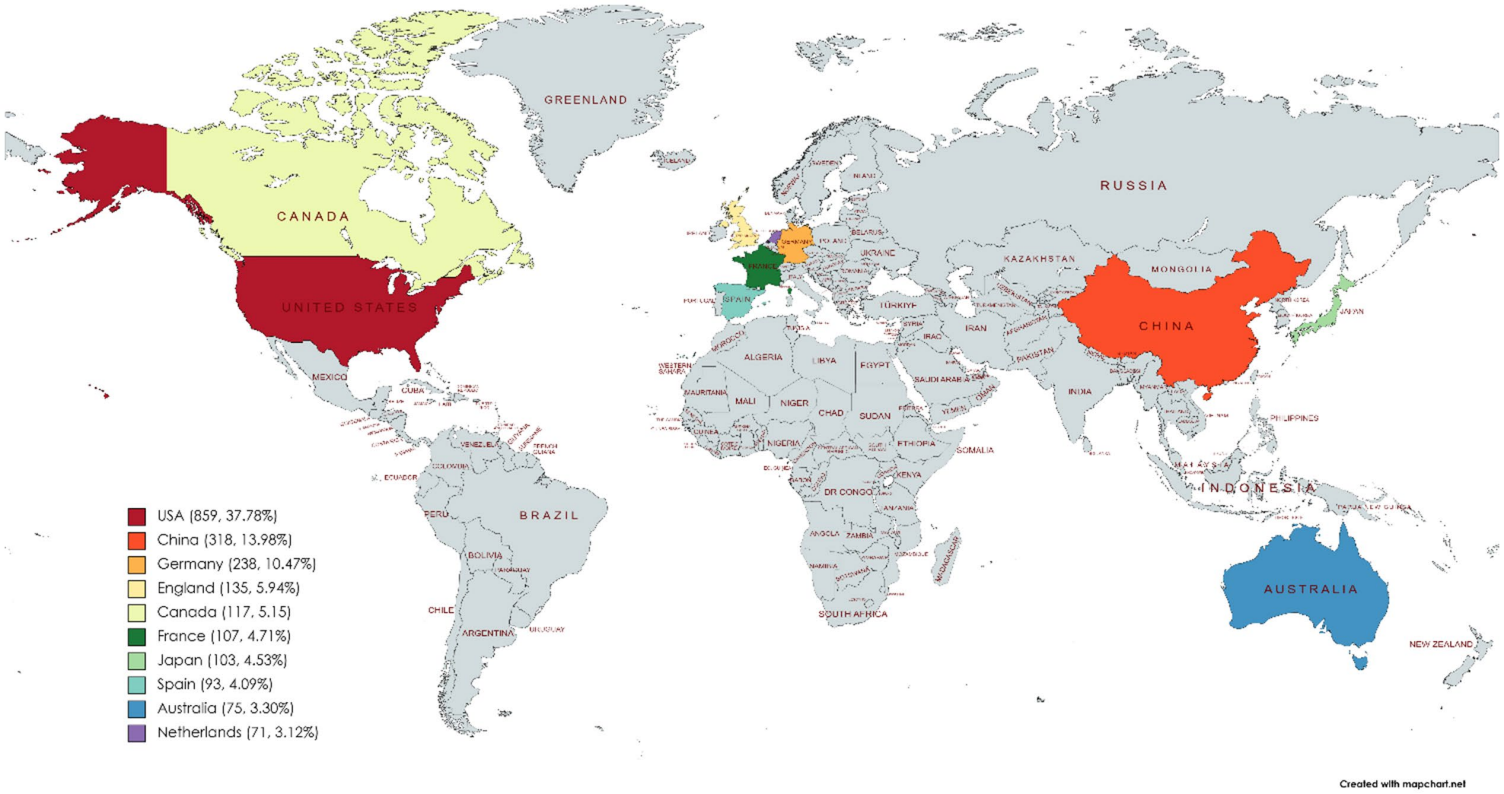
Country/region distribution of MHSI. The map was generated using the web link: https://mapchart.net/world.html.

The number of publications issued by a country in MHSI can serve as an indicator of the level and scale of research conducted in this field, thereby indirectly reflecting the depth and significance of its scientific research. Amongst the 2,274 articles retrieved from the WOS core collection database, a total of 73 countries/regions were involved, including prominent nations such as the United States, China, Germany, England and Canada. This study presents the Top 10 countries/regions based on their publication count, as illustrated in Figure 2. The United States took the lead in terms of the number of articles published in MHSI, with a total of 859 articles accounting for 37.78% of all retrieved articles. China ranked second, contributing 318 publications which accounted for 13.98% of the total. Germany and England followed with 238 and 135 articles, respectively. Canada, France, and Japan have comparable publication rates with each country having published over 100 articles.

**Figure 3:**
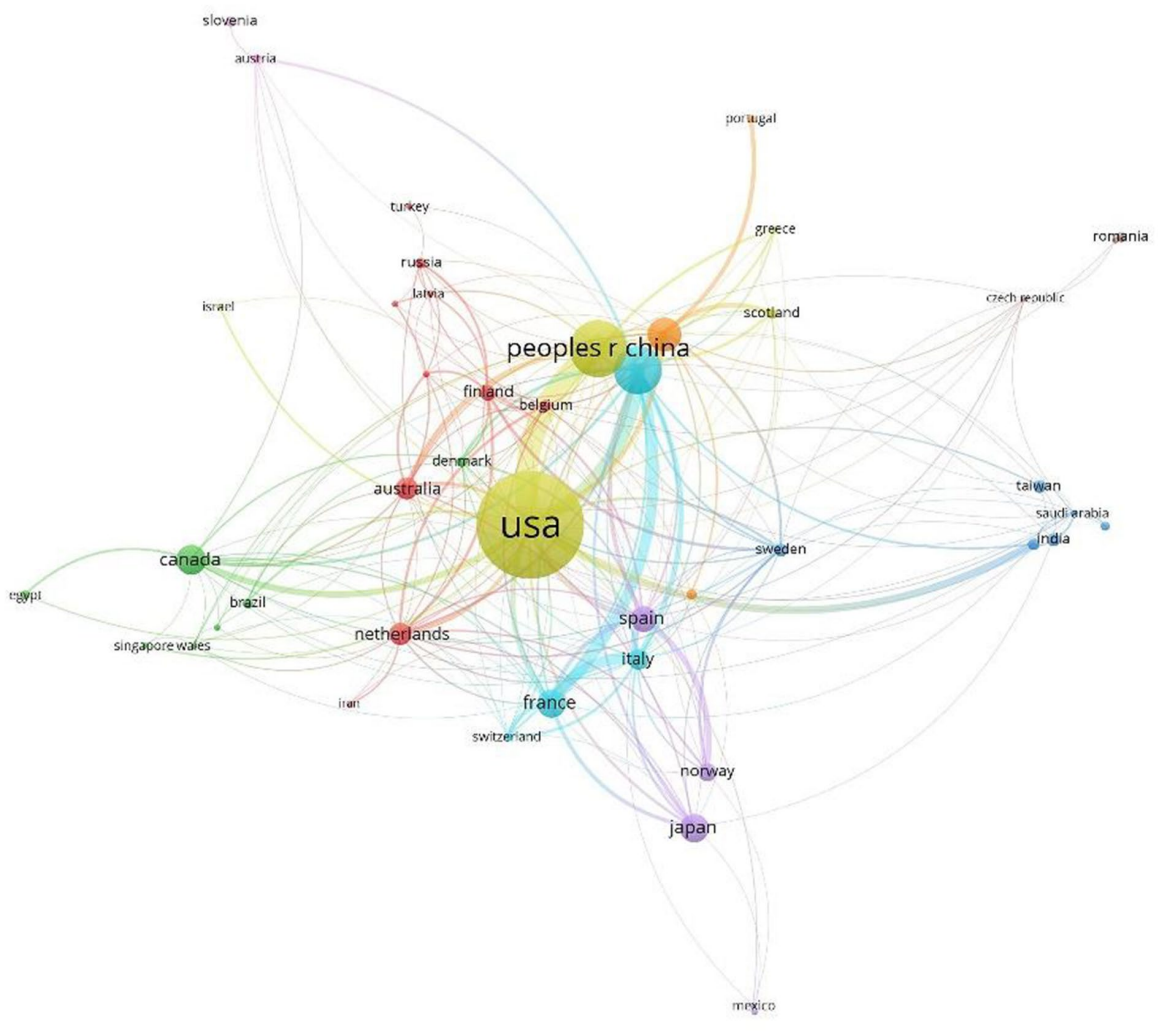
International cooperation in the field of MHSI.

The cooperation relationships between publishing countries/regions in this field are visually analyzed and presented in Figure 3, where the size of the dots represents the number of publications, color indicates clusters, the connections denote cooperation relationships, and segment length reflects the closeness of collaboration among countries/regions. As depicted in the figure, each country has engaged in close cooperation in MHSI.

#### 3.1.3. Core author distribution

The analysis of “Core Author Distribution” facilitates the discovery of leading researchers in the field. Statistics on the number of publications and citations by authors in MHSI, it was found that a total of 7,401 authors are involved. According to Price Law: $M=0.749{\left(N_{\text{max}}\right)}^{1/2}$ , M represents the minimum number of publications by core authors, and $N_{\text{max}}$ represents the number of publications of the most prolific authors in this period (41, 42). It can be concluded that the core authors in this field are those who have published more than six papers, with a maximum of 283 individuals identified as such. Table 1 presents the leading nine authors who have published papers in this study. American Scholar Fei, B.W. and Chinese scholar Li, Q.L. have been the most prolific authors in this field, published 53 articles each, accounting for 2.33% of the total. Gockel, I. a German scholar ranked third with 46 publications, accounting for 2.02% of the total. Scholars Leavesley, S.J. and Rich, T.C., hailing from the United States, hold the fourth and fifth ranks with 43 and 41 published papers, respectively. Other prolific authors include Callico, G.M., Fabelo, H., Ortega, S. and Jansen-Winkeln, B., all of whom have made significant contributions to this area.

**Table 1:** Author distribution in the field of MHSI.

Rating	Author	Country/region	Count (No.)	Contribution (%)	CC
1	Fei BW	United States	53	2.33	2,473
1	Li QL	China	53	2.33	1,075
3	Gockel I	Germany	46	2.02	521
4	Leavesley SJ	United States	43	1.89	275
5	Rich TC	United States	41	1.80	230
6	Callico GM	Spain	40	1.76	858
6	Fabelo H	Spain	40	1.72	858
8	Ortega S	Spain	39	1.63	814
9	Jansen-Winkeln B	Germany	37	1.50	443

**Figure 4:**
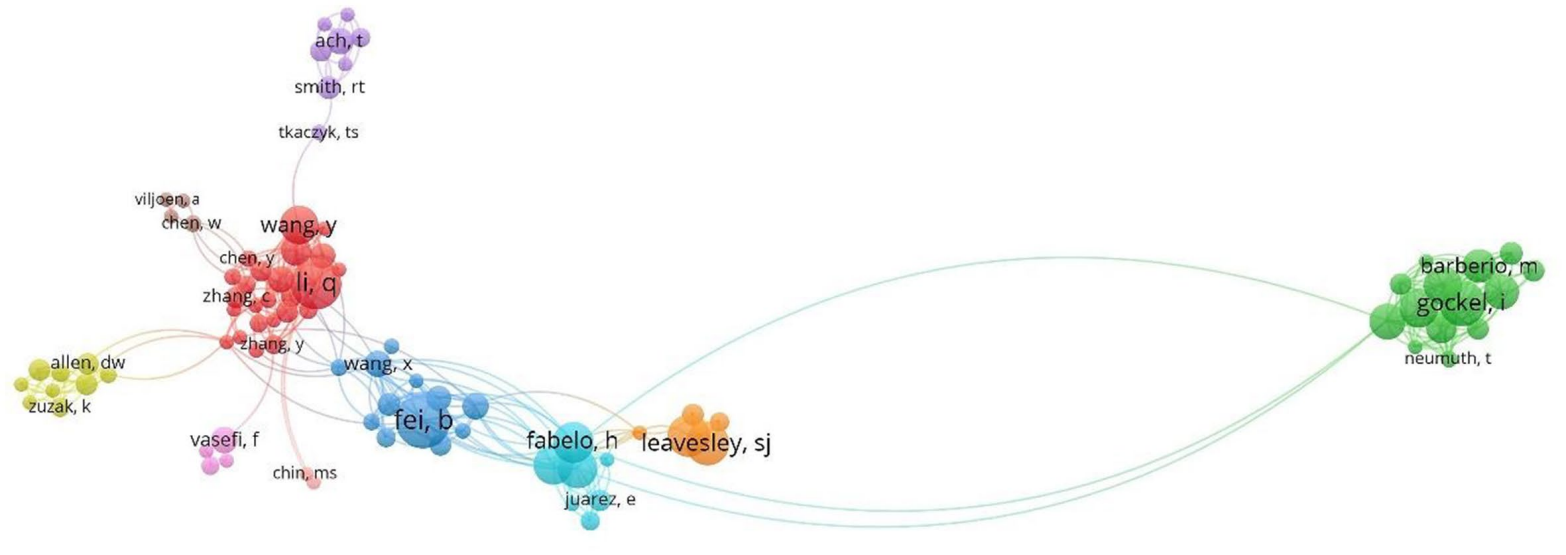
Author collaboration relationships in the field of MHSI.

By filtering each author in WOS database and utilizing the VOS viewer to display the results (Figure 4), it can be found that Lu and Fei’s publication “Medical hyperspectral imaging: a review” from 2014 has been cited an impressive 1,207 times, effectively supporting advancements and progressions within this field while also benefiting numerous interested scholars. Additionally, scholar Fei, B.W. and Spanish scholars Callico, G.M., Fabelo, H., and Ortega, S. engaged in exchanges and collaborations, resulting in numerous joint publications. Furthermore, close collaboration has been established among German scholars Gockel, I. and Jansen-Winkeln, B., as well as American scholars Leavesley, S.J. and Rich, T.C.

#### 3.1.4. Institutional distribution

Bibliometrics analysis enables the identification of leading research institutions in the field, thereby facilitating an understanding of the frontier research and its primary directions. The distribution of researchers can also be identified, with a total of 2,037 institutions involved in the publications retrieved in this study. These include University of Texas System, University of California and University of Texas Southwestern Medical Center at Dallas in the United States, Leipzig University in Germany, East China Normal University in China, and so on. Among these institutions, 19 published more than 30 articles. Table 2 presents the Top 10 according to publication count. Among them, University of Texas System ranked first with 80 articles. Following closely is University of California with 67 articles. The University of Texas Southwestern Medical Center at Dallas, the University of Leipzig, and East China Normal University have respectively, published 63, 55, and 52 articles. In terms of CC, Emory University secured the top position among all institutions in the TOP 10, garnering a total of 2,772 citations. It was followed by University System of Georgia with a CC of 2,512, and the article with the highest CC in this study were authored by these two institutions. Overall, all institutions have shown full research interest in MHSI technology, and the research findings were outstanding.

**Table 2:** Distribution of research institutions in the field of MHSI.

Rating	Institution	Country/region	Count (No.)	CC	BC
1	University of Texas System	United States	80	1,195	0.01
2	University of California	United States	67	1,380	0.06
3	University of Texas Southwestern Medical Center at Dalla	United States	63	929	0.09
4	Leipzig University	Germany	55	689	0.10
5	East China Normal University	China	52	986	0.06
5	Emory University	United States	52	2,772	0.07
7	Udice French Research Universities	France	48	705	0.04
7	University of South Alabama	United States	48	283	0.01
9	Chinese Academy of Sciences	China	42	221	0.04
9	University System of Georgia	United States	42	2,512	0.02

**Figure 5:**
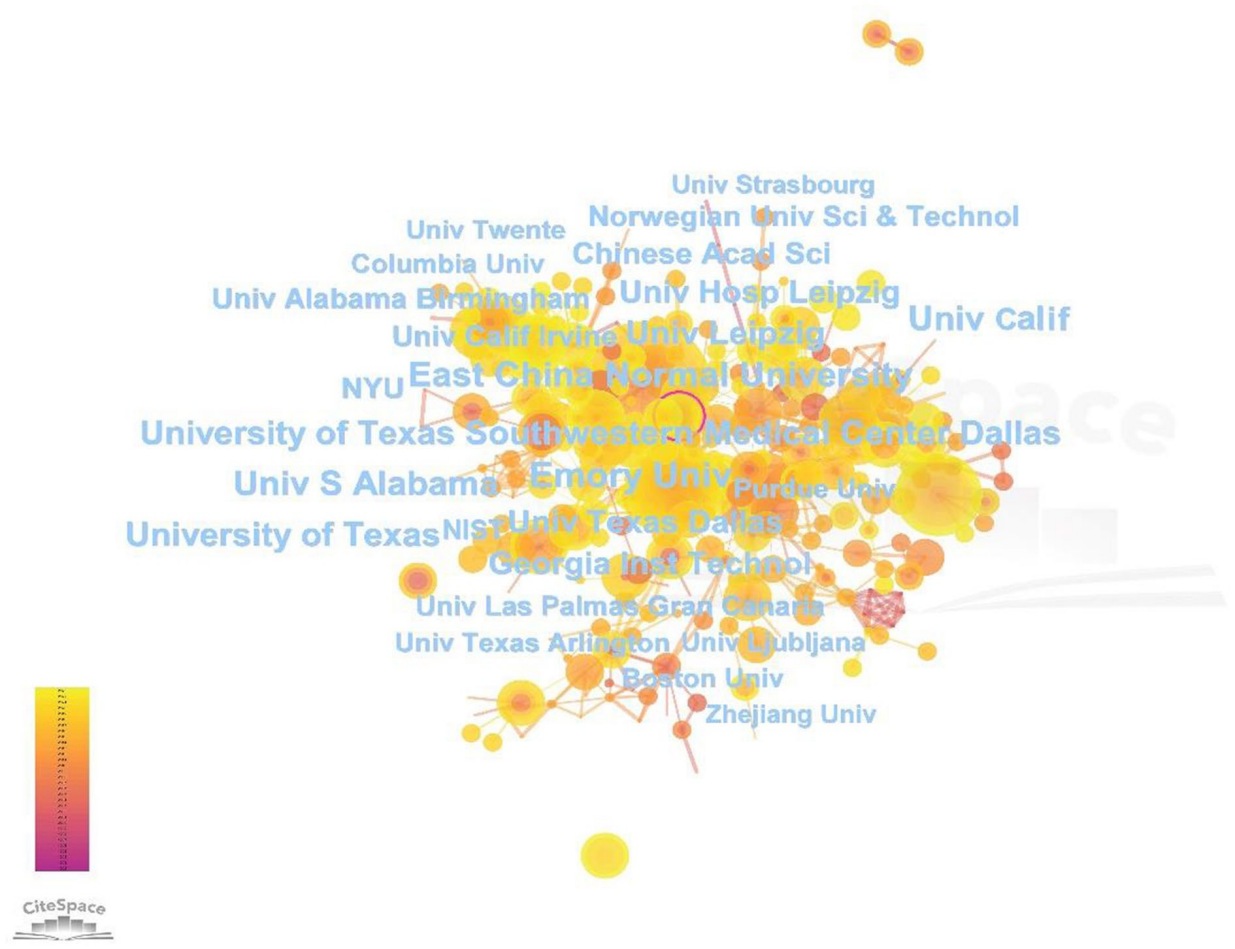
Map of research institutions in the field of MHSI.

Figure 5 displays the map of primary institutions in the MHSI field. Similarly, the larger the node size, the greater its contribution to literature quantity. The node circle represents BC, and nodes with a BC exceeding 0.1 are considered central nodes. Amongst the Top 10 institutions with published articles, Leipzig University (0.10), University of Texas Southwestern Medical Center at Dalla (0.09), Emory University (0.07) exhibited high centrality. In addition to the data presented in Table 2, it is worth noting that Universidad de Las Palmas de Gran Canaria also exhibits a high BC value of 0.14.

#### 3.1.5. Journal distribution

The analysis of published journals can assist scholars in selecting articles, and the impact factor (IF) of the journal to which the article belongs can indirectly indicate the quality of the article, thereby demonstrating researchers’ contribution ability in this field. In addition to choosing journals for submission, scholars also exhibit their accomplishments in certain conferences and books. The 2,274 literatures retrieved in this study encompassed a total of 1,038 journals/conferences. As shown in Table 3, there is a total of 9 journals/conferences with more than 20 articles, among which the Top 3 are Proceedings of SPIE, Journal of Biomedical Optics and Biomedical Optics Express. The proceedings of SPIE published in MHSI totaled 490, accounting for 21.55%. The Journal of Biomedical Optics (IF = 3.758, 2022), a publication under SPIE, contributed 127 articles, accounting for 5.59%. The publications by Biomedical Optics Express accounts for 4.57%, with an impact factor of 3.562. Other journals/conferences such as Investigative Ophthalmology&Visual Science and Proceedings of the Society of Photo Optical Instrumentation Engineers (SPIE) have also gained popularity among researchers in the field of MHSI.

**Table 3:** Distribution of published journals/conferences in the field of MHSI.

Rating	Journal	Count (No.)	Contribution (%)	IF, 2022
1	Proceedings of SPIE	490	21.55	/
2	Journal of Biomedical Optics	127	5.59	3.758
3	Biomedical Optics Express	104	4.57	3.562
4	Investigative Ophthalmology&Visual Science	64	2.81	4.925
5	Proceedings of the Society of Photo Optical Instrumentation Engineers (SPIE)	32	1.41	/
6	IEEE Engineering in Medicine and Biology Society Conference Proceedings	25	1.10	/
7	Sensors	23	1.01	3.847
8	Forensic Science International	21	0.92	2.2
9	Cancers	20	0.88	5.2
10	Computational Intelligence and Neuroscience	19	0.84	3.12

According to the research direction classification in WOS database, it is evident that Optics, Radiology Nuclear Medicine Medical Imaging, Engineering are the primary research areas in MHSI (Table 4). Among them, scholars specializing in Optics have published the most papers with a total of 778 documents—accounting for over one-third of all publications at 34.21%. Radiology Nuclear Medicine Medical Imaging and Engineering ranked second and third, with 627 and 577 documents, respectively. The distribution of literature in different research directions shows the huge diversity of disciplines in MHSI technology, which was a popular research object in many disciplines.

**Table 4:** Distribution of research directions in the field of MHSI.

Research direction	Count (No.)	Contribution (%)
Optics	778	34.21
Radiology Nuclear Medicine Medical Imaging	627	27.57
Engineering	577	25.37
Biochemistry Molecular Biology	308	13.54
Computer Science	191	8.40
Imaging Science Photographic Technology	163	7.17
Surgery	141	6.20
Ophthalmology	131	5.76
Neurosciences Neurology	102	4.49
Pharmacology Pharmacy	94	4.13

#### 3.1.6. Citation analysis

**Figure 6:**
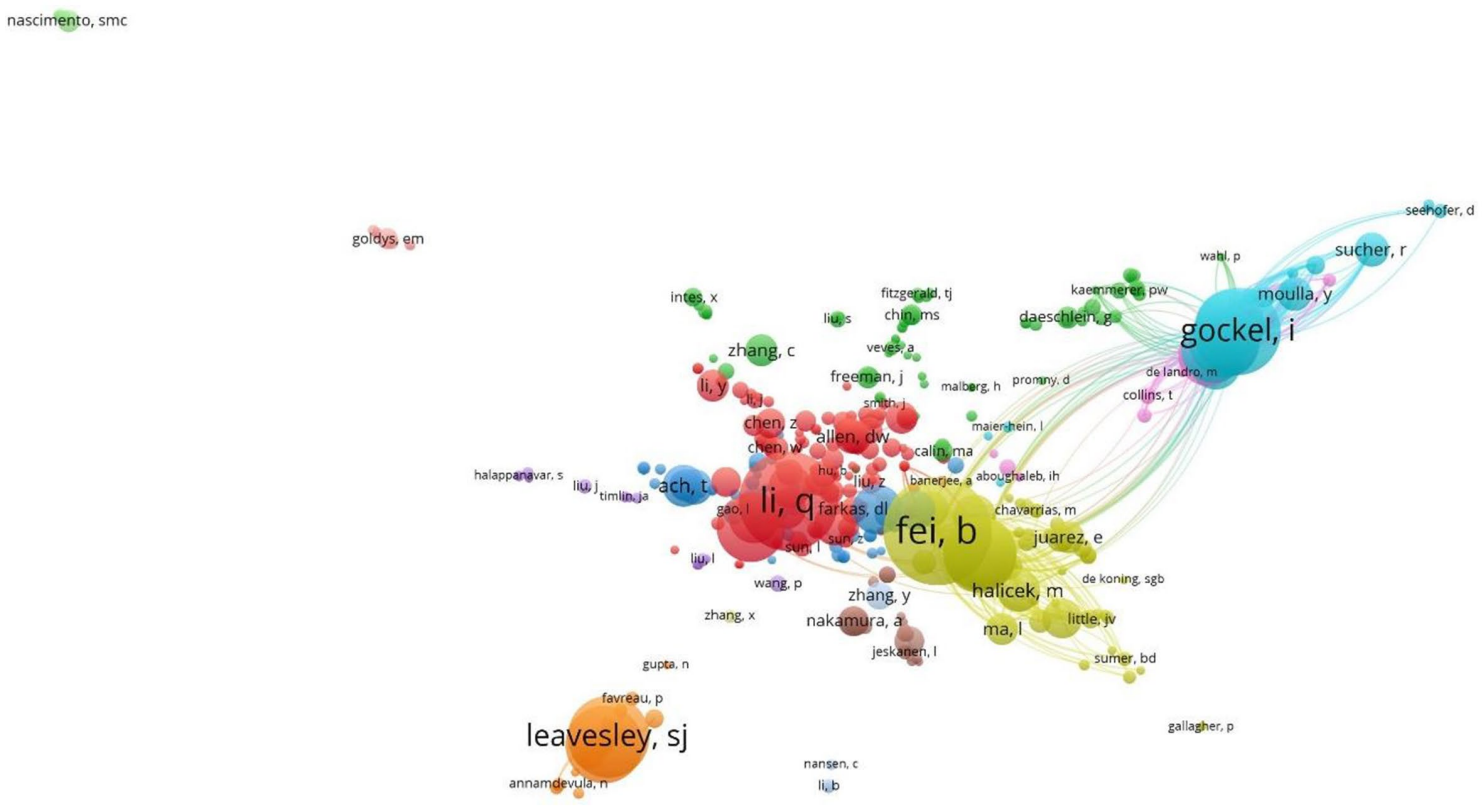
Citation map of hyperspectral imaging technology applications in the medical field.

Citation analysis of scientific literature can effectively characterize its influence and importance, revealing the internal connections between them. A higher CC and CF value indicates greater recognition in the field, as well as providing valuable ideas and directions for scholars’ research. Among the literature collected in this study, 48 articles have a CC exceeding 100 times, with Table 5 listing the Top 10. The most frequently cited publication is “Medical hyperspectral imaging: a review” authored by Lu, G.L. in 2014, which has accumulated 1,207 citations with an annual CF of 134.11, surpassing other research findings significantly. Additionally, the publications by Zibulevsky, M. and Li, J.J. have also received substantial citations—490 and 326 times, respectively, and with the CFs of 22.27 and 54.33 times annually. The relationship among authors with high CC is illustrated in Figure 6, where the size of each node represents its corresponding CC value. Through the distance between nodes, it can be found that, most of the literature searched in this study has a close relationship, and they collectively provide guidance and direction for scholars working in the field of MHSI.

**Table 5:** High cited articles in the field of MHSI.

Title	First author	Journal	CC
Medical hyperspectral imaging: a review	Lu (15)	Journal of Biomedical Optics	1,207
Blind source separation by sparse decomposition in a signal dictionary	Zibulevsky (43)	Neural Computation	490
Lipid desaturation is a metabolic marker and therapeutic target of ovarian cancer stem cells	Li (44)	Cell Stem Cell	326
Modern trends in hyperspectral image analysis: a review	Khan (45)	IEEE Access	320
Hyperspectral and multispectral bioluminescence optical tomography for small animal imaging	Chaudhari (46)	Physics in Medicine and Biology	238
Review of spectral imaging technology in biomedical engineering: achievements and challenges	Li (14)	Journal of Biomedical Optics	234
Hyperspectral imaging of hemoglobin saturation in tumor microvasculature and tumor hypoxia development	Sorg (47)	Journal of Biomedical Optics	230
Portable Spectroscopy	Crocombe (48)	Applied Spectroscopy	216
Real-time imaging of *de novo* arteriovenous malformation in a mouse model of hereditary hemorrhagic telangiectasia	Park (49)	Journal of Clinical Investigation	207
Surface-enhanced Raman scattering for medical diagnostics and biological imaging	Vo-Dinh (50)	Journal of Roman Spectroscopy	189

#### 3.1.7. Keywords analysis

**Figure 7:**
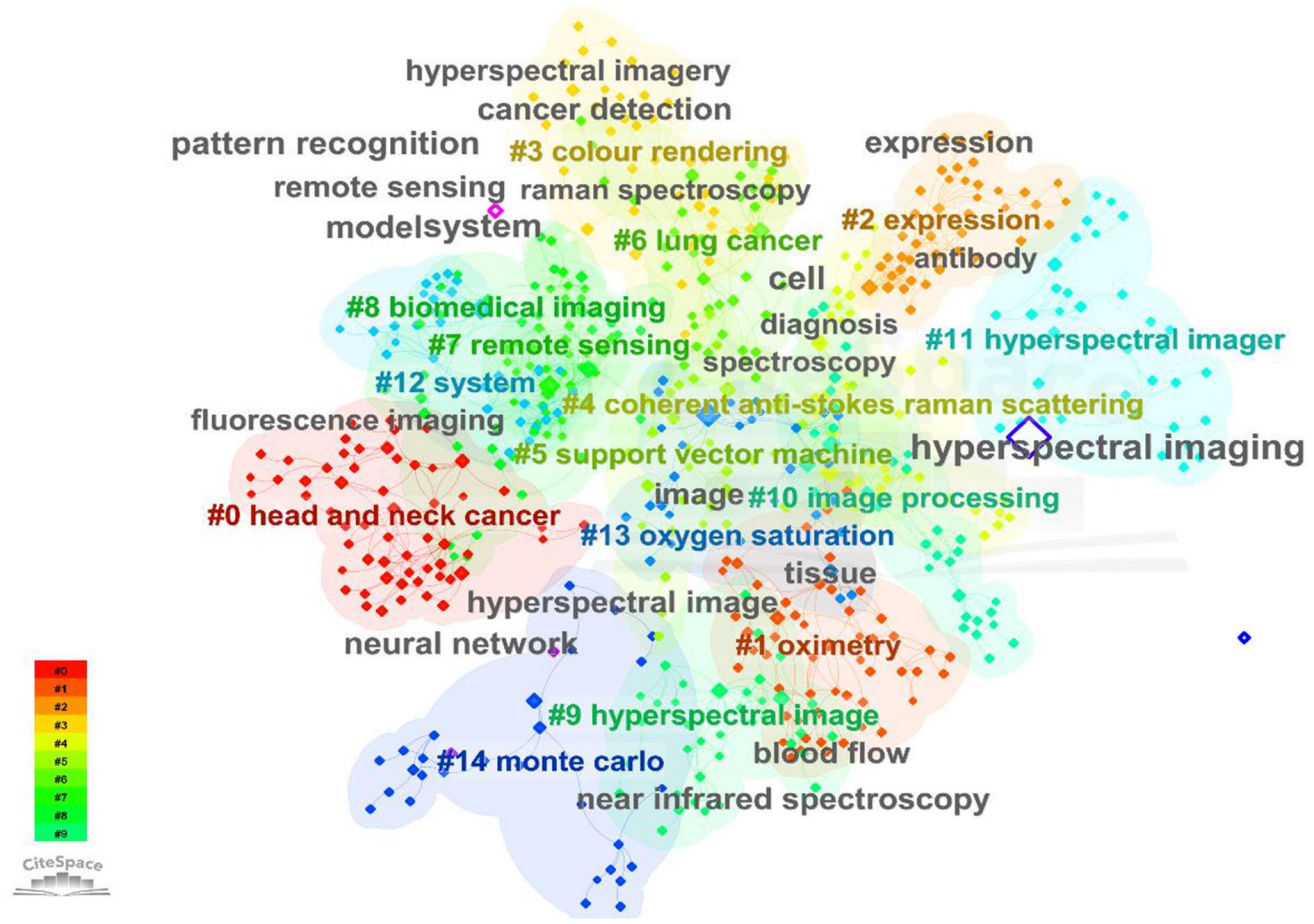
A schematic representation of co-occurring analysis of keywords appeared in the field of MHSI.

**Figure 8:**
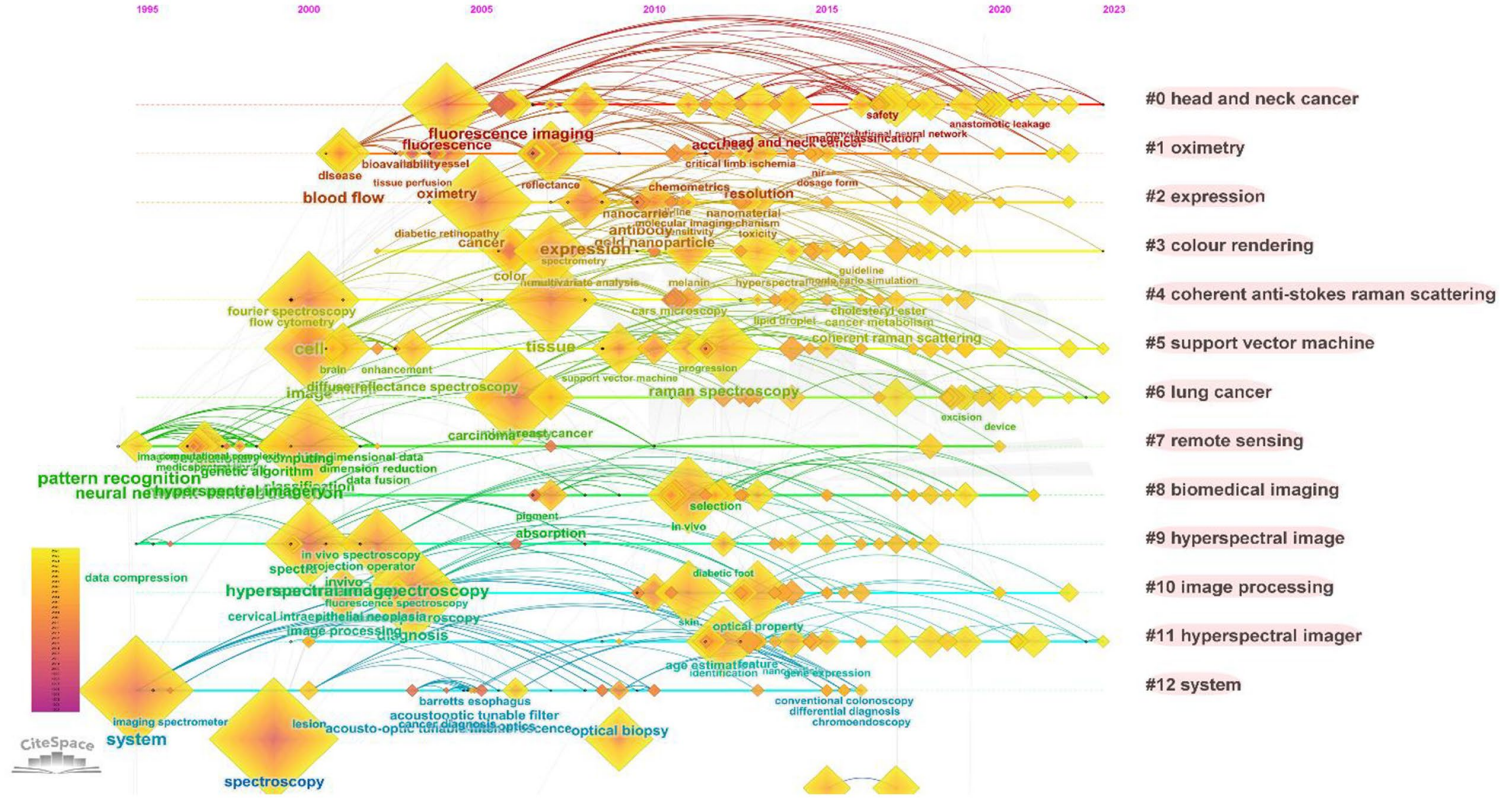
Keyword timeline distribution map.

Keywords provided by the author offer a concise and refined summary of the main content and research direction of an article. Analyzing keyword co-occurrence can reveal research frontiers, hotspots, and development trends in a field. In this study, 7,522 keywords were retrieved from the literature, with 217 appearing more than 10 times. The co-occurrence of keywords was analyzed using CiteSpace with minimized overlap, and the results are shown in Figure 7. The most frequent words in the MHSI field were “hyperspectral imaging,” which was 689 times, followed by “spectroscopy,” “hyperspectral,” “classification,” “microscopy,” “system” and “fluorescence.” Keywords in this field were time-sliced through bibliometrics (Figure 8), revealing that “artificial neural network” burst from 1995 (burst strength 2.32). This trend of exploration has continued until the present day, making it the keyword with the longest duration of burst. Similarly, the time spans of other keywords can also be seen from Figure 8. Among the 7,522 keywords mentioned above, “deep learning” has the highest strength of burst, which is 10.82 (burst from 2021), and for the second “machine learning,” it’s 10.36 (burst from 2019). They have all been research hotspots in recent years.

As depicted in Figure 1, the literature on MHSI obtained from the WOS core database has undergone two primary stages: 1992–2009 and 2010–present. Visual analysis of keywords at each stage can reveal research focal points during different periods, which are presented in Table 6. From 1992 to 2009, there were a total of 325 articles with 1,260 keywords, among which 11 keywords appeared more than 10 times, including “spectroscopy,” “fluorescence,” “hyperspectral,” “hyperspectral imaging,” “microscopy,” “imaging,” and so on. From 2010 to present, some emerging hot words have emerged. A total of 1,949 literature and 6,827 keywords have been retrieved, with 194 keywords appearing more than 10 times, including “hyperspectral imaging,” “spectroscopy,” “classification,” “hyperspectral,” “microscopy,” “cancer,” “deep learning” and so on.

**Table 6:** High frequency keywords at different development stages (frequency ≥ 10 times).

Stage	Keywords
Slow development stage (1992–2009)	Spectroscopy, Fluorescence, Hyperspectral, Hyperspectral Imaging, Microscopy, Imaging, Spectral Imaging, System, Medical Imaging, Classification, Remote Sensing
High-speed development stage (2010–present)	Hyperspectral, Microscopy, System, Cancer, Skin, Diagnosis, Fluorescence, *In-vivo*, Near-infrared Spectroscopy, Cells, Deep learning, Optical Properties, Machine learning, etc.

Figure 7 categorizes the keywords into clusters, revealing 13 clusters, from which we can get that the research hotspots of MHSI mainly focus on “head and neck cancer” (cluster#0), “oximetry” (cluster#1), “expression” (cluster#2), and so on. Cluster#0 contains keywords such as “fluorescence,” “surgery,” “image guided surgery,” etc., cluster #1 contains “reflectance,” “disease,” “resolution,” etc., and cluster #2 contains “cancer,” “gold nanoparticle,” “expression,” and so on. The analysis of keyword clusters and literature content reveals that the primary focus of the article publications can be categorized into two major levels based on different emphases: (1) applied technology; (2) Scope oriented.

### 3.2. Research content in MHSI

#### 3.2.1. Applied technology analysis

Applied technology mainly refers to how HSI technology applies to the medical field, which can be divided into hardware and software aspects. From the retrieved literature, the hardware aspect of MHSI predominantly refers to the system architecture, including overall structure, component composition, as well as design, production, and innovation of internal components. The software utilized in MHSI primarily consists of algorithms, encompassing algorithms and models developed for specific medical research objects, such as machine learning and the subsequent development of deep learning, which have garnered significant attention in recent years (51, 52). These research points ultimately converged into the classification and recognition of medical objects.

**Figure 9:**
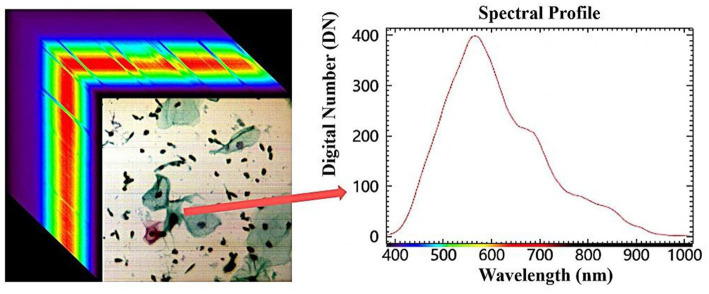
Hyperspectral data cube.

Firstly, the hardware aspect must be considered. HSI technology integrates optical imaging and spectral analysis to produce a three-dimensional (3-D) data cube of spatial and spectral information by collecting spectral information for each pixel of a two-dimensional (2-D) detector array (Figure 9). Through spatial information, spectral information of the region of interest can be obtained to explore its interaction with medicine (15). HSI systems are mainly composed of light sources, dispersion devices and detectors. The light from the light source irradiates the tissue sample, and the information generated is projected into the entrance slit through the front lens, and after collimation, the light is divided into a series of narrow spectral bands by the dispersion device (such as prisms, gratings, etc.), and finally focused onto the detector array. According to the acquisition method, HSI can be divided into whiskbroom HSI, pushbroom HSI and staring HSI. At the same time, according to the different spectral ranges, it can also be divided into ultraviolet (UV), visible (VIS), near-infrared (NIR) and mid-infrared (MIR) HSI systems. The spectral ranges most commonly utilized in the literature retrieved in this study were within the visible and near-infrared regions. Nowadays, HSI have been integrated with various other technologies, including microscopy, laparoscopy, fundus cameras, etc., and the convergence of these technologies highlights their advantages. The most common prevalent combination is that of microscopic hyperspectral imager and fluorescence hyperspectral imager (15). Microscopic hyperspectral imagers are capable of acquiring spectral information from minute substances, and can even identify unstained sections of nervous tissue (53). Fluorescence hyperspectral imagers utilize the inherent fluorescence characteristics of the substance to obtain its fluorescence spectra. All of them provide effective scientific and technological support in the medical field.

From a software perspective, the primary objective is to analyze collected images and extract valuable information. Analyzing medical hyperspectral data at cellular, tissue, and molecular levels can facilitate auxiliary diagnosis, making it an essential component in disease screening, diagnosis, and other processes. Image analysis generally comprises two main components: preprocessing and data analysis (54). Preprocessing involves enhancing image quality and simplifying algorithms before data analysis, including image segmentation, data normalization and image filtering, etc. (55). This serves to mitigate the impact of background noise on sample spectral information while eliminating errors stemming from baseline drift, surface scattering, or noise introduced by uneven lighting or dark current in the lens (56), as hyperspectral images inevitably introduce noise information during the acquisition process, which will restrict the accuracy of image analysis (57). Data analysis involves the utilization of support vector machine (SVM), k-nearest neighbor (KNN), artificial neural network (ANN), and other algorithms which combines both spectral and spatial features. This has been demonstrated by Ravikanth et al. (58) and Yan et al. (59) as well as Wang et al. (60). Simultaneously, owing to the high similarity between adjacent bands in hyperspectral data, it becomes imperative to employ dimensionality reduction techniques for extracting the most pertinent information and reducing dataset dimensions. Principal component analysis (PCA) and successive projections algorithm (SPA) are widely utilized methods in this regard.

In summary, the construction of hardware and the analysis of software, as crucial components of HSI, will significantly impact its practical application in the medical field. The breakthroughs in key technologies such as advanced spectroscopic technology and high frame rate detector technology will enhance the hardware capabilities of HSI and improve information acquisition proficiency (38). Therefore, achieving higher spectral resolution, spatial resolution, and sensitivity is the goal of scholars. With the deepening of algorithm research related to HSI, the adaptability of algorithm model to medical research goals is very important. On the basis of traditional machine learning algorithms such as SVM (61) and K-means clustering (62), the rise of deep learning provides new ideas for the processing of medical images (63). A large number of studies are also endeavoring to enhance the recognition accuracy and sensitivity of specific medical objects through changing model parameters or structures.

#### 3.2.2. Scope oriented analysis

Scope orientation mainly refers to the application of hyperspectral imaging technology in the medical field. From the retrieved literature, it can be seen that the research subjects encompass various aspects of the medical field, primarily medical imaging diagnosis and medicine research. Medical imaging diagnosis encompasses cell detection, blood analysis, skin and tongue examination, tumor identification, cancer screening, image-guided surgery and more (64–67). Medicine research involves the sourcing and quality control of medicinal materials, analysis of drug counterfeiting as well as the detection of pesticide residues and types (68).

When conducting measurements on the reflection and absorption processes of light at different wavelengths, HSI can provide information on different tissue components and their spatial distribution. Tissues in distinct pathological states exhibit varying reflectivity at specific wavelengths due to disparities in their chemical composition and physical characteristics, resulting in distinctive spectral features such as hemoglobin, melanin, and water that selectively absorb light across different wavelengths ranges. Based on these characteristic spectral signals, qualitative and quantitative analysis of the tissues can be achieved, and combined with spatial information, visual identification of the organization can be completed. It will provide a strong basis for organizational identification. Zherdeva et al. (69) used HSI technology to visualize skin tumors and confirmed the possibility of distinguishing melanoma at different growth stages, which provided basis and guidance for real-time identification of malignant melanoma. Kiyotoki et al. (70) successfully distinguished gastric tumor samples from normal samples using a VIS-HSI system (400–800 nm), with specificity and sensitivity of 92.5% and 78.8%, respectively. Fabelo et al. (71) used HSI technology to develop a real-time high-precision detection device for brain tumors during surgery, and found that it provided effective help in tumor resection surgery by neurosurgeons, accurately determined the boundaries of tumors, and the accuracy of preliminary experiments exceeded 95%. Optical absorption can also react to the enhancement of angiogenesis and metabolic activity by quantification of hemoglobin and oxygen saturation concentrations. Chin et al. (72) used a VIS-HSI system to detect peripheral arterial disease (PAD), and found that the lower limb oxygenation level of PAD patients and normal people showed differences, which can be used for diagnosis and evaluation of patients with PAD. Jeffcoate et al. (73) analyzed tissue oxygenation HSI data in diabetic patients and showed that HSI technology can predict early healing in diabetic foot ulcer patients.

The authenticity and quality control of traditional Chinese medicine are crucial for the advancement of Chinese medicine and national healthcare. HSI technology has been successfully applied to identify the origin and mold changes of various Chinese medicinal materials, such as peach and apricot kernels (74), ginseng (75), and agaricus bisporus (76). In addition, HSI technology enables both qualitative and quantitative analysis of medicinal materials, pesticide residues, etc. (77). Scholars have successfully employed HSI technology to visualize flavonoid content in Ginkgo biloba leaves, as well as analyze loquat leaves rapid determination of triterpenoid acids (78, 79).

From this, it is evident that HSI technology has a broad range of object-oriented applications in the medical field. It can not only achieve comprehensive imaging of biological tissues and organs, microscopic imaging of pathological sections, but also plays a crucial role in medical detection, igniting a new wave of technological advancement in the medical field.

## 4. Discussion

### 4.1. Research status

In the past three decades, HSI technology has made significant strides and is now an indispensable tool in various fields, including military, agriculture, medical care, and daily life. The application of MHSI aligns with the trend of digitalization, scientific advancement, and intelligence in the medical field by meeting evolving medical needs while enhancing quality to establish a new model for medicine.

Based on the WOS core database, this study conducted a visual analysis of 2,274 publications in the field of MHSI retrieved from 1992 to present. Through scientific bibliometric methods, we systematically sorts out the distribution of country/regions, core authors, institutions, journals, citations, keywords, etc., and the development trends and research hotspots in this field were also obtained.

In the initial stage (1992–2009), HSI technology underwent rapid development and its application gradually expanded, starting to be applied in medical fields. Medical hyperspectral data was developed, leading to a series of explorations in areas such as forensics, damaged tissue detection, health assessment and variant tissue resolution. In the second stage (2010-present), research enthusiasm has continued to surge, with a year-on-year increase in publications (the study was conducted in July, 2023, which still has room for improvement), showing a J-shaped curve growth, indicating that the field is developing rapidly and the research has not yet reached saturation. In addition, MHSI technology is being integrated with the constantly evolving technology in modern times. Machine learning algorithms, such as deep learning, have been effectively employed for feature extraction and classification recognition of hyperspectral images (16, 80). Moreover, the research field has continuously expanded its scope to encompass the classification and recognition of various other subjects including cancer, tumor, and skin (64, 81–83), forming a positive development trend that keeps pace with the times.

The United States, China, Germany, England, Canada, etc. have conducted extensive research in this field. All of them have attached great importance to it and demonstrated their enthusiasm for research. Among them, the United States, Germany, England, etc. have closer cooperation with China while Japan has established close connections with Spain and Norway, etc. Enhanced collaboration has been established among nations. In terms of the issuance of documents by the top 10 institutions, the United States holds six positions, and the most CC literature also originates from there. The majority of articles in the field of MHSI were authored by American Scholar Fei, B.W. and Chinese scholar Li, Q.L., Scholar Fei, B.W., who is affiliated with University of Texas at Dallas and University of Texas Southwestern Medical Center, authored the highest-rated review article on CC in this study. He has conducted extensive research primarily based on the microscopic identification of tumors and cancers, such as head and neck squamous cell carcinoma and aggressive papillary thyroid carcinoma, and aims to explore the application of HSI technology in image-guided surgical procedures. He has collaborated closely with Spanish scholars Callico, G.M., Fabelo, H., and Ortega, S. All of them are among the top nine authors and have jointly published numerous achievements. It can be observed that there are eight collaborations with Universidad de Las Palmas de Gran Canaria, which demonstrate a closely-knit cooperative relationship. Spanish researchers Callico, G.M., Fabelo, H., Ortega, S., all affiliated with Universidad de Las Palmas de Gran Canaria. These authors have maintained a close collaboration, also resulting in numerous co-authored publications. The research outcomes of this team using HSI technology to conduct rich explorations on medical objects such as brain cancer, brain tumors, skin, melanoma, gastroenterology etc., and continuously explore algorithms to improve target recognition, such as PCA, SVM, and artificial intelligence (AI). In recent years, a series of studies have been conducted on medical equipment based on HSI technology. Chinese scholar Li Q.L., affiliated to East China Normal University, has conducted extensive research in the field of MHSI since the year 2006 (based on 2,274 samples retrieved in this article). In the early stages, a series of studies were conducted on tongue analysis in traditional Chinese medicine using HSI technology. In recent years, research has mainly been conducted on automatic classification recognition and related algorithms at the pathological or cellular level using microscope hyperspectral. Scholars have made significant advancements in this field, driving progress forward. Above all, it is evident that HSI technology is garnering increasing scholarly attention and has demonstrated significant research value in the medical field. Simultaneously, similar to the research directions of the aforementioned scholars, AI technology is being employed in MHSI to achieve automatic recognition of medical objects, presenting promising prospects for further development.

Despite extensive research in the field of MHSI, certain limitations still exist. Hardware constraints, such as spectroscopic devices and detectors, can impact information acquisition quality and require ongoing development. At the same time, selecting suitable software algorithms for the research of specific medical targets from a large number of options is challenging, and new algorithm models are constantly emerging, leading to some confusion in their development. Additionally, there exists a certain barrier between HSI technology and medical disciplines, and the vast amount of knowledge introduced by MHSI requires specialized expertise. Therefore, the field of MHSI still presents numerous challenges.

In our research, while the utilization of the WOS core database has facilitated access to a substantial number of literatures in the MHSI field, providing a broad overview of the current research landscape, there is still room for improvement. Firstly, the WOS database is widely recognized as one of the most authoritative and comprehensive document retrieval databases available. Additionally, EI Compendex, Scopus, etc. are also commonly utilized document retrieval database worldwide. It should be noted that there are variations in the literature included within each respective database. For this study, only one database was selected, it may benefit from combining multiple databases to more comprehensively reflect information within the field in future research. Furthermore, among the 2,274 articles included in this study, only a small number are written in German, Chinese and French, resulting in a scarcity of local language literature in the field of MHSI across different countries. In addition, this study utilizes the TypeScript (TS) retrieval method and employs the search term “hyperspectral,” with a query scope primarily encompassing titles, abstracts, and keywords. Although this study has included a significant number of medical articles utilizing HSI technology through category screening, there may still be a few articles that cannot be incorporated if the TS content lacks the search term “hyperspectral.” Therefore, when utilizing Bibliometrics for research purposes, it is imperative to explore more effective sample selection methods, expand the scope of literature coverage within the field, enhance compatibility and ultimately provide a more comprehensive reflection of both progress and shortcomings in said field.

### 4.2. Future research directions in MHSI

In an era of heightened health concerns, HSI technology has evolved into an intelligent and information-based tool for enhancing diagnostic efficiency, promoting objectivity, and better serving the needs of patients seeking testing equipment. As technical capabilities continue to mature and research expands its scope, it’s imperative that we redouble our efforts to fully integrate HSI technology into medical practice.

a) In addition to the overall structural design of the HSI system, achieving higher spectral resolution, spatial resolution and sensitivity of components are the key to technological development and imaging quality. These challenges pose opportunities for researchers as their implementation will significantly enhance the application of MHSI technology. Furthermore, HSI technology not only provide spectral information in the visible range but also in the ultraviolet, near-infrared, and mid-infrared regions. Exploring these additional spectral ranges allow for the extracting of unique features that cannot be found within the visible region, which may play a significant role in expanding medical research objects.

b) The algorithms applicable to a specific medical field are different, and a plethora of diverse algorithms have been utilized in existing publications, making it arduous to establish a standard. In the future, selecting appropriate HSI systems and data processing algorithms for specific research fields and establishing standards to augment their applicability towards more medical objects will be the development trajectory of MHSI.

c) The continuous improvements of instrumentation, software design, and algorithmic developments in spectral imaging technology will make the analysis results of medical objects more complex (14). Nowadays, it is expected that more researchers and doctors try this technology in their biomedical research. In the future development, it is necessary to cultivate more interdisciplinary talents to establish the connection between instrumentation and medicine.

Providing more optical feature information for the medical field, alleviating the shortage of doctors, and facilitating seamless integration between medicine and HSI technology. It will be of great significance to develop MHSI technology. Anticipating a substantial leap forward in the practical application of MHSI technology!

## 5. Conclusion

The bibliometrics method employed in this study offers scholars a scientific reference to comprehend the developmental process and research content of the entire MHSI field, while also highlighting areas for improvement in existing research. The bibliometric results demonstrate that MHSI technology is extensively researched worldwide and holds great potential as an effective tool to aid doctors. Currently, the development of MHSI technology is generally positive, but it still faces certain challenges that require collaborative efforts from scholars to effectively integrate it into medical daily life.

## Author contributions

SJ, DM, and XT contributed to conception and design of the study. SJ organized the database and wrote the first draft of the manuscript. DM performed the statistical analysis. DM, XT, QJ, MY, and LX wrote sections of the manuscript. All authors contributed to the article and approved the submitted version.

## Funding

This work was supported by the National Natural Science Foundation of China (NSFC) (61975199); Capital construction funds in Jilin Province in 2023 (2023C036-4); Changchun Science and Technology Development Plan Project (22SH03 and 21SH17); Jilin Province Science and Technology Development Plan Project (20220201060GX); Jilin Province and Chinese Academy of Sciences Science and Technology Cooperation High Tech Industrialization Special Fund Project (2023SYHZ0020); Jilin Province Database of Agriculture Spectrum Application Information (20230505009ZP).

## Conflict of interest

The authors declare that the research was conducted in the absence of any commercial or financial relationships that could be construed as a potential conflict of interest.

## Publisher’s note

All claims expressed in this article are solely those of the authors and do not necessarily represent those of their affiliated organizations, or those of the publisher, the editors and the reviewers. Any product that may be evaluated in this article, or claim that may be made by its manufacturer, is not guaranteed or endorsed by the publisher.
